# ROS induces NETosis by oxidizing DNA and initiating DNA repair

**DOI:** 10.1038/s41420-021-00491-3

**Published:** 2021-05-18

**Authors:** Dhia Azzouz, Meraj A. Khan, Nades Palaniyar

**Affiliations:** 1 Program in Translational Medicine, Peter Gilgan Centre for Research and Learning, The Hospital for Sick Children, Toronto, ON Canada; 2 Department of Laboratory Medicine and Pathobiology, University of Toronto, Toronto, ON Canada; 3 Institute of Medical Sciences, Faculty of Medicine, University of Toronto, Toronto, ON Canada

**Keywords:** Immune cell death, DNA mismatch repair

## Abstract

Reactive oxygen species (ROS) are essential for neutrophil extracellular trap (NET) formation or NETosis. Nevertheless, how ROS induces NETosis is unknown. Neutrophil activation induces excess ROS production and a meaningless genome-wide transcription to facilitate chromatin decondensation. Here we show that the induction of NADPH oxidase-dependent NETosis leads to extensive DNA damage, and the subsequent translocation of proliferating cell nuclear antigen (PCNA), a key DNA repair protein, stored in the cytoplasm into the nucleus. During the activation of NETosis (e.g., by phorbol myristate acetate, *Escherichia coli* LPS, *Staphylococcus aureus* (RN4220), or *Pseudomonas aeruginosa*), preventing the DNA-repair-complex assembly leading to nick formation that decondenses chromatin causes the suppression of NETosis (e.g., by inhibitors to, or knockdown of, Apurinic endonuclease APE1, poly ADP ribose polymerase PARP, and DNA ligase). The remaining repair steps involving polymerase activity and PCNA interactions with DNA polymerases β/δ do not suppress agonist-induced NETosis. Therefore, excess ROS produced during neutrophil activation induces NETosis by inducing extensive DNA damage (e.g., oxidising guanine to 8-oxoguanine), and the subsequent DNA repair pathway, leading to chromatin decondensation.

## Introduction

NETosis is a unique form of programmed cell death that neutrophils undergo when exposed to certain agents such as phorbol myristate acetate (PMA), bacterial LPS, *Staphylococcus aureus* (RN4220), or *Pseudomonas aeruginosa*^1–5^. These agonists activate NADPH oxidase (NOX), which generates reactive oxygen species (ROS) and subsequently activates mitogen-activated protein kinases (extracellular signal-regulated kinase, p38, c-Jun N-terminal kinase)^6–10^. Khan and Palaniyar have recently shown that a genome-wide transcriptional firing initiated by the activation of kinase cascade is necessary to decondense chromatin and drive NETosis^11^. They have shown that transcription initiation, but not mRNA translation and new protein synthesis, is essential for NETosis. The assembly of transcription machinery is one of the key factors that facilitates chromatin decondensation at promoter regions. However, the relevance of DNA repair on NETosis is unknown.

It is apparent that NOX-dependent NETosis agonists induce the generation of massive amounts of ROS in neutrophils^1–3,6,12,13^. In fact, ROS is essential for NETosis because inhibiting ROS production by pharmaceutical (e.g., diphenyliodonium or DPI) or genetic (e.g., mutations in NOX subunits) inhibition of NOX activity completely inhibits NOX-dependent NETosis^2^. Nevertheless, how ROS executes NETosis remained unknown.

We hypothesised that ROS induces DNA damage, and repairing extensive DNA damage by DNA repair leads to the full opening of chromatin and subsequent NETosis. ROS could oxidise the bases of DNA (e.g., converting guanine to 8-oxoguanine). When transcription machinery stalls at the damaged locations of the DNA, repair machinery assembles on those sites and opens chromatin for repair^14–17^. Damaged bases could also be repaired independently of the transcription machinery. These include 8-Oxoguanine glycosylase (OGG1), proliferating cell nuclear antigen (PCNA), apurinic/apyrimidinic endonuclease (APE) 1, poly-adenosine diphosphate (ADP) ribose polymerase (PARP), DNA ligase, and DNA repair polymerases (pol) β and δ^18–21^. Various pathways exist to repair damaged bases (e.g., base excision repair (BER), or nucleotide excision repair (NER)^17^). To initiate BER DNA repair, the oxidised base is removed by a DNA glycosylase, which is followed by strand cleavage by APE1. Next, PARP binds to the single stranded DNA ends at the cut sites and generates poly ADP, often attaches these polymers to PARP itself or to the nearby histones. These events recruit the rest of the repair machinery, including DNA ligases. Depending on the nature of the damage (non-bulky or bulky lesions), PCNA forms a trimeric ring around the DNA duplex and allows the repair DNA polymerases β and δ to interact with the PCNA clamp and other repair protein complexes to complete the DNA repair (long-patch BER or NER)^17^. Assembly of the first three enzymes at the damaged sites (APE1, PARP, DNA Ligase) and nick generation is sufficient to induce extensive chromatin decondensation at these loci^22^. Therefore, in this study, we tested the importance of ROS-mediated DNA damage and each key step of the DNA repair pathway in driving chromatin decondensation and subsequent NET formation (see graphic abstract).

## Results

### ROS generated by NOX induces extensive DNA damage

**Fig. 1:**
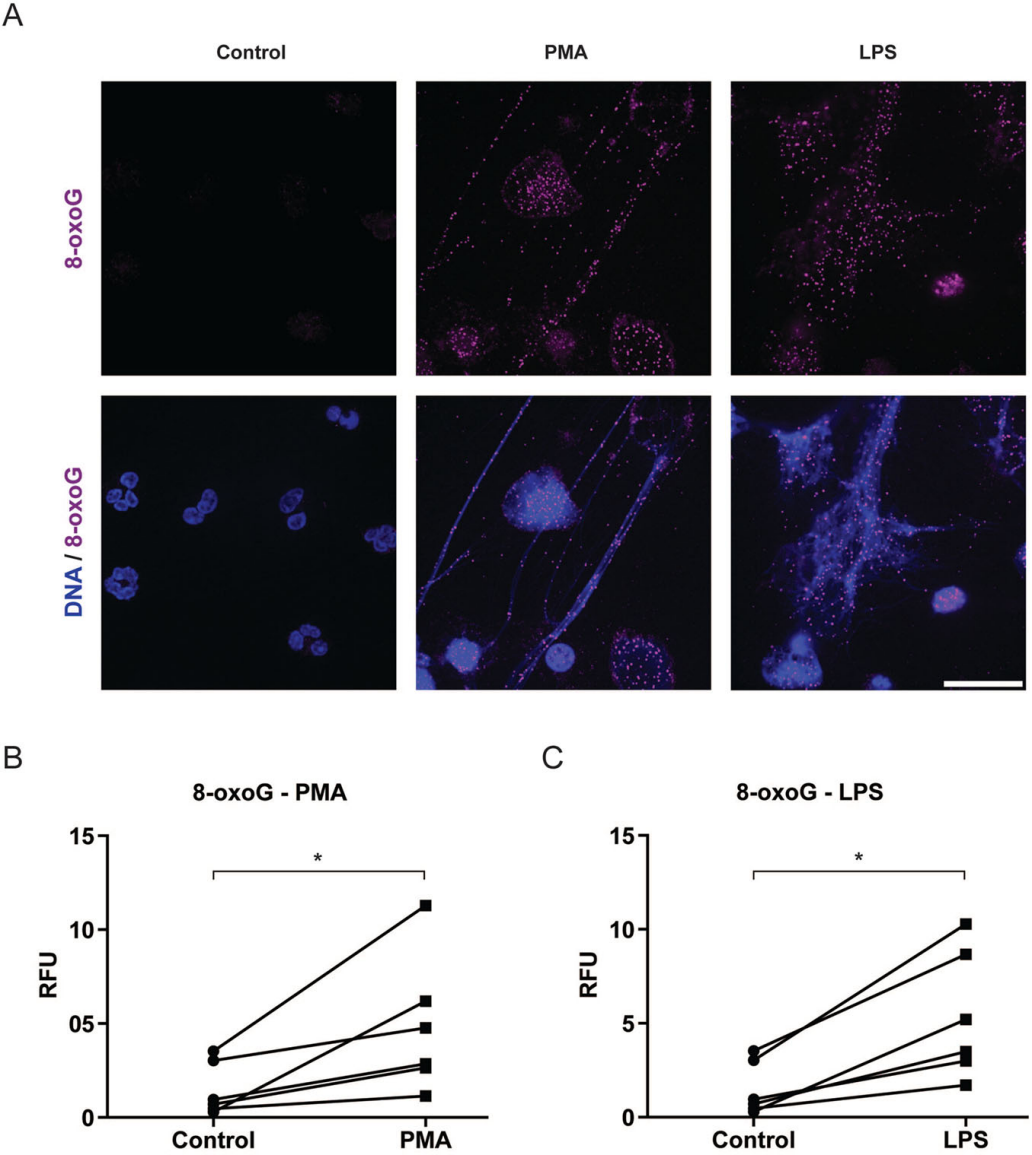
PMA- and LPS-treated neutrophils generate increased levels of 8-oxoG. **A** Neutrophils were treated with PMA (25 nM) or LPS (5 μg/ml) and incubated for 240 min. Cells were stained for DNA (DAPI, blue) and 8-oxoG (purple). The images are representative of three independent experiments. Scale bar, 10 μm. **B-C** 8-oxoG levels following treatment with media (-ve control), PMA (25 nM) or LPS (5 μg/ml) were measured using an in-cell ELISA. LPS- and PMA-treated cells had significantly greater 8-oxoG levels compared to media treated cells (*n* = 6; *p < 0.05).

The primary endogenous DNA damaging agent is ROS, which oxidises DNA bases. Oxidation damage to purine results in various chemical modifications to the ring atoms. One of the most common and deleterious modifications is the formation of 7,8-dihydro-8-oxoguanine (8-oxoG)^23^. To determine the effect of ROS in DNA damage, we first induced NETosis by two well-characterised NOX-dependent ROS generating agonists, PMA and LPS. To determine the extent of DNA damage, 8-oxoG, the primary product of ROS-induced DNA damage, was studied using immuno-confocal imaging. The images showed the presence of massive amounts of oxidative damage throughout the NET DNA (Fig. 1A). We quantified the 8-oxoG content by an in-cell ELISA. The in-cell ELISA results corroborate the images and showed that significantly high amounts of 8-oxoG are present in neutrophils following the induction of NETosis using PMA or LPS (Fig. 1B).

### Cytoplasmically stored DNA repair protein PCNA translocates into nuclei and binds to DNA during NETosis

**Fig. 2:**
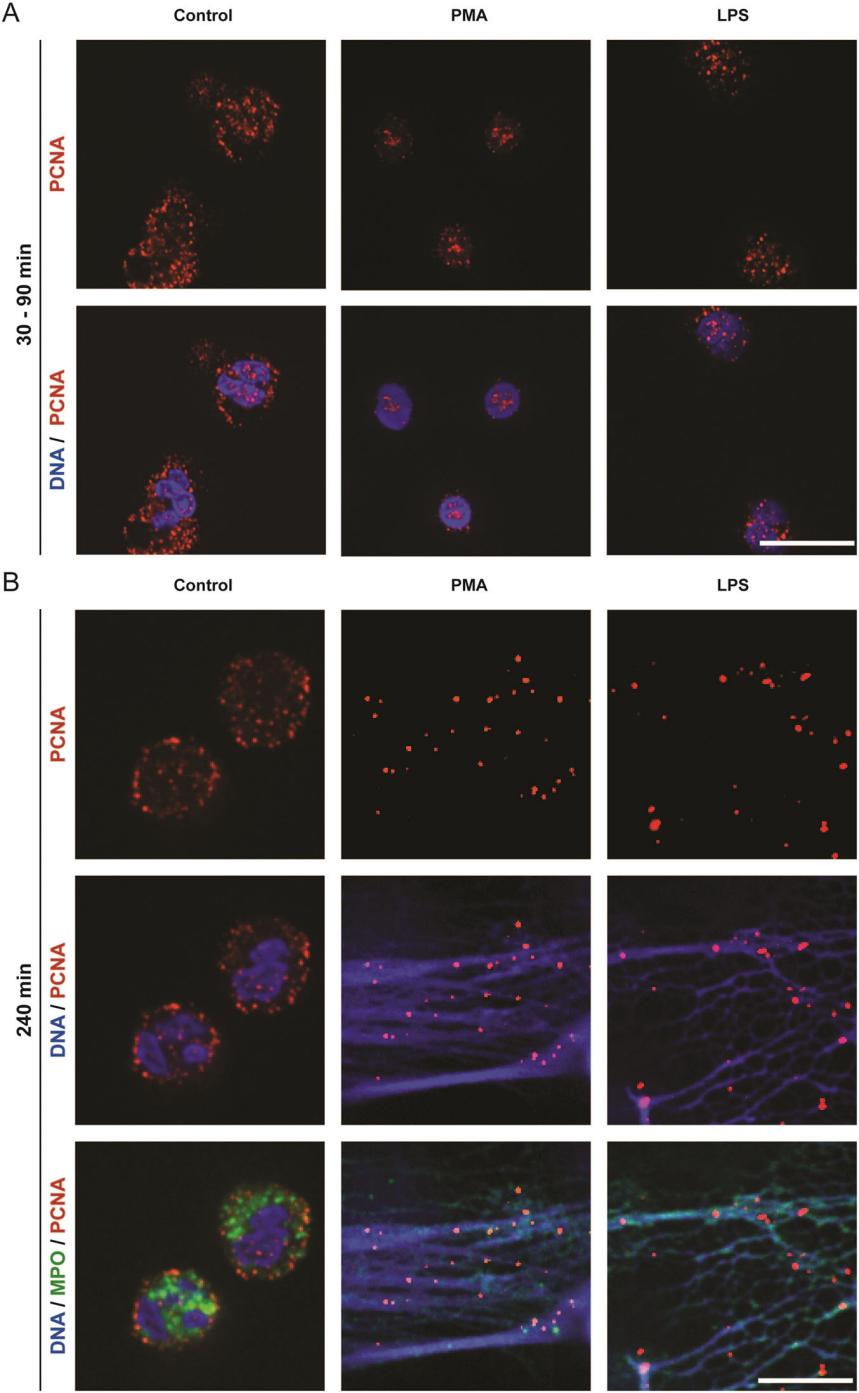
PCNA translocates into the nucleus/perinuclear regions following the induction of NETosis with PMA or LPS, and eventually decorates the NET DNA. **A** Neutrophils were treated with PMA (25 nM) or LPS (5 μg/ml) and incubated for 90 or 30 min, respectively. Cells were stained for DNA (DAPI, blue) and PCNA (red). **B** Neutrophils were treated with PMA (25 nM) or LPS (5 μg/ml) and incubated for 240 min. Cells were stained for DNA (DAPI, blue), PCNA (red) and MPO (green). The images are representative of three independent experiments. Scale bar, 10 μm.

Since extensive DNA damage was occurring in PMA- or LPS-treated neutrophils, DNA repair could be involved in NETosis. BER or NER pathways are primarily responsible for repairing oxidative damage. In healthy neutrophils, PCNA is present in the cytoplasm; PCNA is a DNA clamp involved in repair pathways, and was proposed as a regulator of neutrophil apoptosis^19^. However, whether PCNA is involved in NETosis was unknown. Therefore, to determine whether PCNA translocates into the nucleus following the induction of NETosis, we studied the PCNA localisation at different stages of NETosis using confocal microscopy. PCNA was present in large quantities in the cytoplasm in unstimulated neutrophils, with little present within the nucleus (Fig. 2A). By contrast, following treatments of neutrophils with PMA for 60–120 min and LPS for 30–60 min, PCNA was exclusively found to localise in the nuclear/perinuclear regions, with little present in the cytoplasm (Fig. 2A). This drastic translocation of PCNA into the nucleus is indicative of the initiation of DNA repair process. Staining for PCNA in cells at the later stages of NETosis (e.g., 4 h post PMA or LPS), uncovered that PCNA was localised throughout the NET DNA (Fig. 2B), indicating that the repair machinery was assembling in many parts of the genome during NETosis. We also confirmed true NET induction by PMA and LPS by staining NETs with myeloperoxidase (MPO), a known NETosis marker that colocalizes with NETs (Fig. 2B). Therefore, DNA repair process is highly active after the induction of ROS (typically peaks ~30-min post PMA or LPS treatment) in neutrophils.

### Inhibitors of early, but not late steps of the repair pathway suppress agonist-induced NETosis

**Fig. 3:**
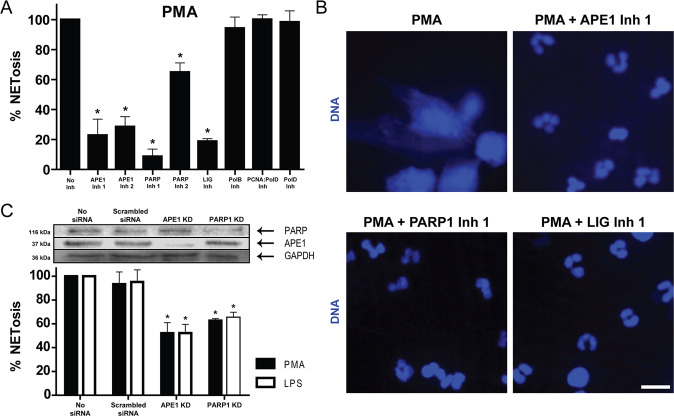
Inhibition of early steps, but not late steps, of DNA repair suppresses NETosis induced by PMA and LPS. **A** DNA release from neutrophils following media (-ve control) or PMA (25 nM) was measured using the SYTOX Green plate reader assay. Cells were preincubated with BER inhibitors (APE inh 1, CRT0044876 (125 μM); APE inh 2, APE1 Inhibitor III (50 μM); PARP1 inh 1, BSI201 (100 μM); PARP inh 2, PJ34 (50 μM); LIG inh, L189 (100 μM); Pol β inh, AM-TS23 (25 μM); PCNA:Pol δ inh, T2AA (25 μM) or Pol δ inh, Aphidicolin (50 μM)) for 1 h prior to PMA treatment (*n* = 3; *p < 0.05 compared to positive control). **B** Neutrophils were incubated with DNA repair inhibitors for 1 h then treated with PMA (25 nM), incubated for 240 min, and stained for DNA (DAPI, blue). The images are representative of three independent experiments. Scale bar, 10 μm. **C** Differentiated HL-60 neutrophils were pretreated with media, a scrambled siRNA, APE1, or PARP siRNA. Cells were then treated with PMA (150 nM) or LPS (15 μg/ml) for 6–8 h. NETosis levels were measured using the SYTOX Green plate reader assay. Western blots were used for verifying knockdown. The data are presented as mean ± SEM (*n* = 4; *p < 0.05 compared to the control with no inhibitors).

To determine the importance of various steps of DNA repair pathway on NETosis, we first performed the SYTOX Green plate reader assays. SYTOX Green is a cell impermeable dye that fluoresces green following its binding to DNA released by neutrophils. Hence, the levels of green fluorescence act as a measure of NETosis. Cells were incubated with BER/NER pathway inhibitors (APE1, PARP1, DNA ligase, PCNA, and polymerases β/δ inhibitors) for an hour prior to treatment with media control or PMA for 4 h. The base removal step was not targeted as different enzymes are involved in the removal of different types of oxidised bases. APE1, PARP1, and DNA ligase inhibitors decreased NETosis following the stimulation of neutrophils with PMA (Fig. 3A). However, inhibitors of PCNA:polymerases β/δ interactions or DNA polymerase β activity failed to reduce PMA-induced NETosis (Fig. 3A). We confirmed that both PCNA and polymerase inhibitors are functional, in different assays (unpublished data). SYTOX Green assay results were confirmed by confocal imaging (Fig. 3B). These studies show that initial chromatin decondensation steps of BER/NER, but not the steps after PCNA binding and DNA polymerase activity are necessary for ROS-mediated NETosis.

### APE1 and PARP1 siRNA silencing resulted in reduced NOX-dependent NETosis in HL-60 cell-derived neutrophils

Primary neutrophils are not amenable for knockdown studies due to the short lifespan of these cells. Therefore, to confirm the inhibitor data obtained in primary neutrophils, we used siRNA-based knockdown in differentiated HL-60 cells. Differentiated HL-60 neutrophils, after knocking down APE1 or PARP1, were treated with media control, LPS, or PMA. Since HL-60 neutrophils require more time for NETosis, we incubated these neutrophils with PMA or LPS treatment, for a longer time (6–8 h) to observe substantial NETosis. NETosis assays showed that the knockdown of APE1 and PAPR1 also resulted in reduced NETosis (Fig. 3C), confirming the overall finding that the early steps of DNA repair are important for agonist-induced NETosis, by another experimental approach.

### The same groups of DNA repair inhibitors also suppress bacteria-induced NETosis

**Fig. 4:**
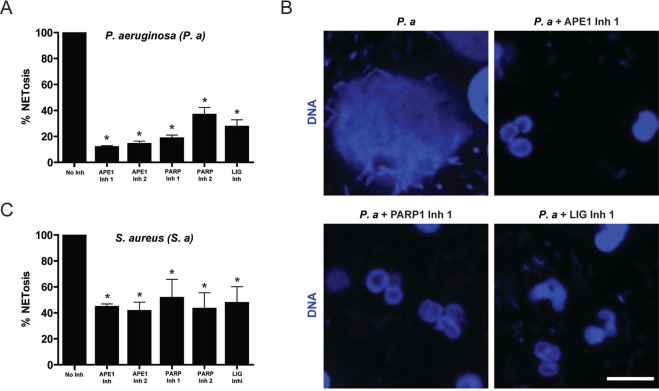
Inhibition of early steps of BER suppresses NETosis induced by *P. aeruginosa* and *S. aureus*. **A**, **C** DNA release from neutrophils following media (-ve control), 20 MOI *P. aeruginosa* or 20 MOI *S. aureus* (RN4220) was measured using the SYTOX Green plate reader assay. Cells were preincubated with BER inhibitors (APE inh 1, CRT0044876 (125 μM); APE inh 2, APE1 Inhibitor III (50 μM); PARP1 inh 1, BSI201 (100 μM); PARP inh 12, PJ34 (50 μM) or LIG inh, L189 (100 μM)) for 1 h prior to bacterial treatment. The data are presented a mean ± SEM (*n* = 3; *p < 0.05 compared to the control with no inhibitors). **B** Neutrophils were incubated with DNA repair inhibitors, that inhibited NETosis, for 1 h then treated with 20 MOI *P. aeruginosa* and incubated for 240 min, and stained for DNA (DAPI, blue). The images are representative of three independent experiments. Scale bar, 10 μm.

In order to provide more biologically relevant data, the ability of the DNA repair inhibitors to suppress *Staphylococcus aureus* or *Pseudomonas aeruginosa* was also studied. Since these two bacteria induce NOX-dependent NETosis^4^, we incubated neutrophils with 20 MOI of *S. aureus* (RN4220) or *P. aeruginosa* for 4 h and the amount of NETs released by the neutrophils was examined by microscopy and quantified using the SYTOX Green plate reader assays. Results indicated that the inhibitors for the three enzymes APE1, PARP1, and DNA ligase also significantly reduced *S. aureus*- and *P. aeruginosa*-induced NETosis (Fig. 4A–C).

## Discussion

**Fig. 5:**
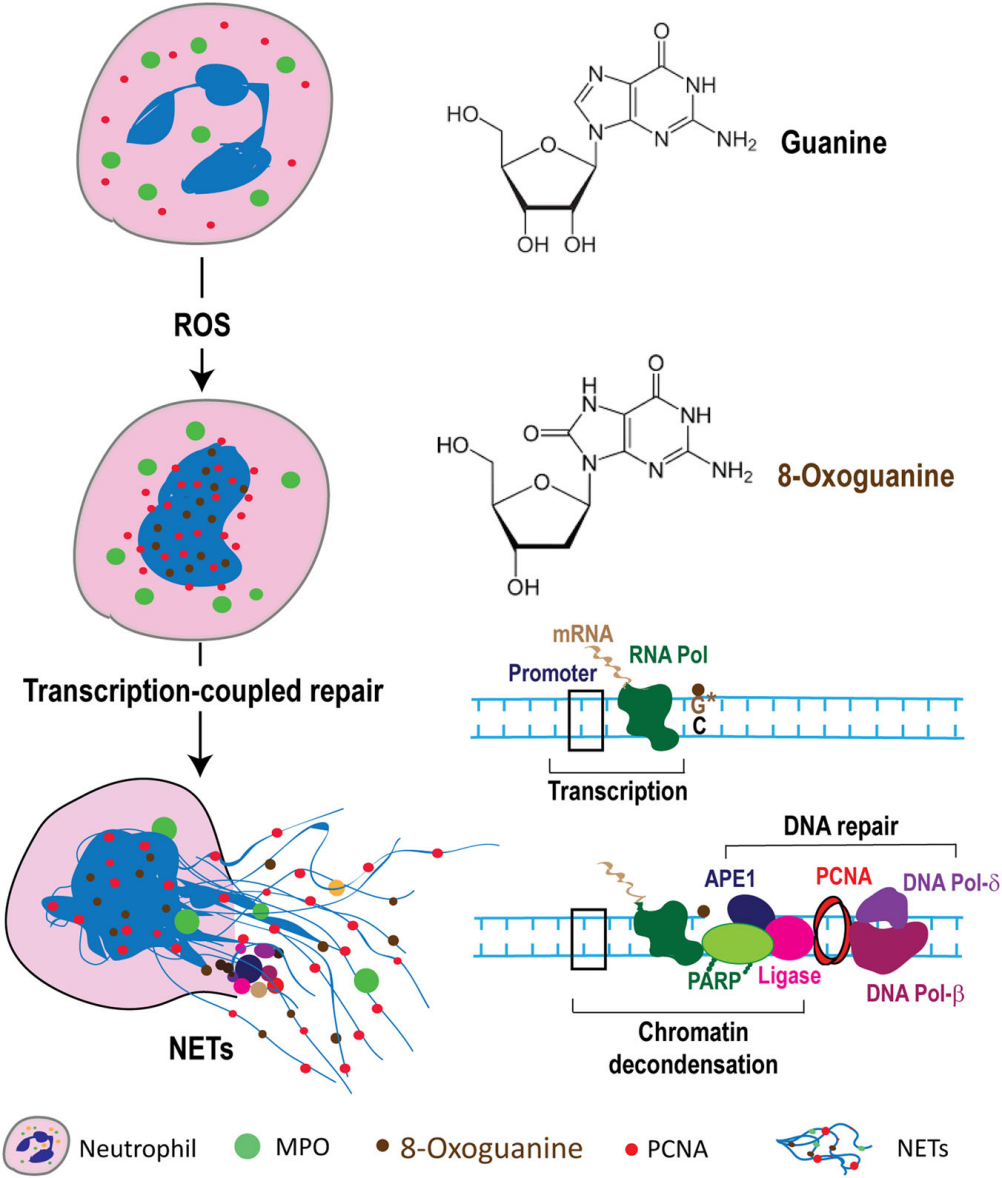
Summary figure. Diagram showing the role of DNA damage repair during NETosis in the context of the other known components of the NETosis pathway.

Neutrophils are terminally differentiated cells that generate large amounts of ROS, and we considered DNA repair in a unique context. ROS generates DNA lesions in the form of modified bases that can impair or stall transcription and DNA replication. Neutrophils do not replicate their DNA, but have evolved to repair DNA damages^24^. We have previously shown that NETosis agonists induce a genome-wide meaningless transcriptional firing during NETosis^11^. Transcription machinery stalls at the sites of damaged DNA and initiates DNA repair^25^. For the first time, our current study has uncovered that the chromatin unwinding capability of the DNA repair machinery is a key driver of NETosis. Therefore, we propose that during NOX-dependent NETosis, ROS first activates various MAPKs cascades leading to activation of transcription factors and subsequent transcriptional firing. In addition, ROS oxidises guanine to 8-oxoguanine. These events recruit DNA repair machinery to the sites of DNA damage and decondenses chromatin. This form of DNA repair provides novel insights as to how ROS induces NETosis (see graphic abstract; Fig. 5).

In this study, we first used NOX-dependent NETosis agonists such as PMA and LPS to show that ROS induces DNA damage. Typically, in response to microbial infections, neutrophils activate NOX and generate ROS in the phagosomes to kill microbial pathogens. However, neutrophils generate large amounts of ROS and generate NETs at high MOI (e.g., >5) of bacteria or bacterial components such as LPS^4,8^. Activated NOX produces O_2_^.−^ which is catalysed by superoxide dismutase to H_2_O_2_, which is then catalysed by MPO to HOCl. H_2_O_2_ can also be converted to OH. Radicals^26^. O_2_^.^^−^, HOCl and OH. radicals oxidise macromolecules, including DNA. The primary product of DNA oxidation by ROS is 8-oxoG^27^. Our studies show that both PMA and LPS induce large amounts of DNA damage in the form of 8-oxoG (Fig. 1).

Neutrophils carry DNA repair proteins including OGG1, PCNA, PARP, and DNA pol β^18–21^. For example, DNA helix clamping protein PCNA is stored in the cytoplasm^19^. Our data clearly show that upon the activation of NOX-dependent NETosis (i.e., ROS, DNA damage), PCNA exclusively localises to the nuclei of these neutrophils (Fig. 2). BER/NER is the primary pathway for removing oxidative lesions, and involves base/nucleotide removal, incision, end processing, repair synthesis, and ligation^17^. PCNA is mainly associated with long-patch repair in BER, and patch NER^28^, suggesting that ROS-mediated damage also involves such types of repairs. By the end of NETosis, PCNA is found associated throughout the NET DNA suggesting that DNA damage (Fig. 1) and repair occurs in all the regions of the genome (Fig. 2). Previous studies suggested that PCNA is stored in neutrophils to regulate apoptosis^19^. We propose that PCNA is stored in neutrophils to also regulate DNA repair and NETosis.

Our results indicate that the outcome of inhibition of the DNA repair pathway depends on the target selected. This effect is seen for NETosis induced by PMA, LPS, *P. aeruginosa*, and *S. aureus* (inhibitors or siRNA knockdown; Figs. 3 and 4). The roles of BER proteins are common to all ROS-induced lesions, not just 8-oxoG; hence, the base removal step was omitted as DNA glycosylases are responsible for removing the bases for different types of ROS-induced lesions. The inhibition of early steps of NETosis (APE1, PARP1, DNA ligase) resulted in suppression of NETosis, while the inhibition of later steps (PCNA, DNA polymerases; these inhibitors are functional as determined by other assays; data not shown) did not reduce agonist-induced NETosis. One potential explanation of the results is as follows: by inhibiting the early steps of the pathway, we are preventing the opening of the chromatin that would have been observed if the repair machinery were allowed to assemble at the site of the damage, and the nicking of the DNA. This is supported by findings uncovered by other research groups that reported that steps prior to DNA ligase recruitment can be performed without any structural disruption to nucleosomes^22^. By inhibiting DNA ligase, the preceding steps such as APE1 activity will still take place but no NETosis will be observed as these steps do not result in the chromatin remodelling. On the other hand, by inhibiting the later steps, we are allowing for the majority of the machinery to form, which involves extensive unwinding of the chromatin^22^. These results suggest that steps up to DNA ligase are essential for NETosis to occur as these steps provide sufficient unwinding and nicking of the chromatin, which is supported by the findings that suggest that inhibitors of later steps of BER do not inhibit agonist-induced NOX-dependent NETosis. Since strong agonists induce excess ROS production that leads to genome-wide DNA damage and subsequent chromatin decondensation by the assembly of initial DNA repair machinery, the later steps become less important under these conditions. However, we cannot exclude the possibility of PCNA, DNA polymerase β, and other DNA repair proteins regulating different stages, or forms of NETosis.

APE1 is a multifunctional enzyme within cells. While the C-terminal is responsible for the DNA endonuclease activity, the N-terminal has redox activity^29^. During oxidative conditions, APE1 has been reported to activate several transcription factors, such as p53 and others^29^. While this begs the question of whether the reduction of NETosis resulting from administration of APE1 inhibitors is likely due to the subsequent inhibition of transcription factor activation, our results suggest otherwise. Inhibition of other early steps of BER provided the same reduction in NETosis, this suggests that the observed reduction in NETosis is due to the inhibition of the endonuclease activity of APE1. Furthermore, the APE1 inhibitor CRT0044876 is known to bind to the active site of APE1 and impair its 3′-phosphodiesterase and 3′-phosphatase activities^30^. As the functions of the two terminals of APE1 are independent of one another, the redox activity of APE1 should not be affected by CRT0044876.

PCNA and polymerase δ are classically associated with long-patch BER whereas polymerase β is associated with short-patch BER. However, PCNA and polymerase δ activity during short-patch BER (1 nucleotide), and polymerase β activity during long-patch BER (2–~13 nucleotides) have been previously reported on several occasions^31–33^. Long-patch BER has been reported to be less important in terminally differentiated cell types, unlike short-patch BER, which suggests that short-patch BER^34^ is mainly responsible for the unwinding of the chromatin during agonist-induced NETosis. Interestingly, DNA replication is not necessary for NETosis. Previous studies also show that DNA replication does not drive NETosis^35^. Therefore, we conclude that the events of DNA repair, but not replication, is involved in driving NETosis.

It has been previously shown that the inhibition of MAPKs, which activate transcriptional factors, results in the inhibition of NETosis^6,8^. Therefore, we see a significant inhibition of NETosis when either MAPK activation or BER are abolished. This suggests that the two chromatin unwinding machineries are meeting in the same pathway. Given that RNA polymerase stalling occurs at sites of DNA damage and that the stalling results in the recruitment of DNA repair machinery^25^, it is logical to surmise that DNA repair is coupled to transcription in the context of NETosis. While transcription-coupled DNA repair machinery assembles at the RNA polymerase stalled sites, Global Genome NER may also assemble the repair machinery on multiple loci on the non-transcribed genomic regions. BER machinery could assemble in multiple locations. Although the relative contributions of specific types of DNA repair pathways in NETosis remain to be established, the genome-wide unwinding process promoted by transcription and repair at multiple sites of DNA damage ultimately results in the complete opening of the chromatin for NETosis.

Classical thinking is that neutrophils carry DNA repair machinery to restore the integrity of their genome after heavy oxidative bursts. The involvement of DNA repair machinery in NETosis uncovered in this study is another example of the participation of the machinery outside of the canonical context of DNA repair and cell-cycle checkpoint. These results assign a novel function to the elaborate DNA repair machinery found in neutrophils that until now, have been considered to not have sufficiently meaningful activity^18^. We propose that neutrophils carry DNA repair machinery to exert a do-or-die attempt to protect themselves and the host. Specific DNA repair pathway inhibitors identified in this study may be used for treating NETosis-related diseases, and to explore potential relevance of these repair proteins in diseases associated with excess NET formation (e.g., Lupus, autoimmune diseases, sepsis).

## Methods and materials

### Ethical clearance

Protocols were approved by the Hospital for Sick Children ethics committee. All methods were performed in adherence with the set guidelines and regulations. Subjects signed informed consent forms.

### Neutrophil isolation from human peripheral blood

Peripheral blood drawn from healthy donors was placed in K2 EDTA blood collection tubes. Neutrophils were isolated as previously published using PolymorphPrep^4^. Modifications to the manufacturer’s neutrophils isolation protocol were made. Red blood cell lysis step was added by using a hypotonic solution of 0.2% (w/v) NaCl. The solution was then made isotonic and buffered by adding an equal volume of 1.6% (w/v) NaCl solution with Hepes buffer (20 mM, pH 7.2). The cells were washed twice with a solution of 0.85% (w/v) NaCl and Hepes (10 mM, pH 7.2). Neutrophils were then resuspended in RPMI medium (Invitrogen) containing Hepes buffer (10 mM, pH 7.2).

### SYTOX Green plater reader assay for NETosis analysis

SYTOX Green dye (5 μM; Thermo Fisher Scientific) was added to cells (5 × 10^5^ cells per ml). Cells were seeded on a 96-well plate. Inhibitors were added to the cells, followed by a 1 h incubation at 37 °C. Media control (negative control), NOX-dependent agonists and Triton X-100 (positive control) were then used as cell activators. The inhibitors used were APE inh 1 (CRT0044876, Sigma), APE inh 2 (APE1 Inhibitor III, EMD Millipore), PARP1 inh 1 (BSI201, Sigma), PARP inh 2 (PJ34, EMD Millipore), and LIG inh (L189, Tocris). They were dissolved in DMSO and diluted in media before being added to the samples at the required concentrations. Fluorescence of SYTOX Green-DNA interaction was measured using POLARstar OMEGA fluorescence plate reader (BMG Labtech; excitation = 485 nm, emission = 525 nm) every 30 min for 240 min Plotted Data represents NETosis levels. NETosis index was determined by dividing the fluorescence reading of each treatment by the reading of 1% (v/v) Triton X-100-treated cells.

### Confocal imaging

Cells (1 × 10^6^ cells per ml) were plated on a 96-well plate, and incubated with inhibitors for 1 h at 37 °C. Following induction of NETosis, reaction proceeded for an allotted amount of time at 37 °C before being terminated with 4% (w/v) paraformaldehyde (PFA; Sigma-Aldrich) overnight. Cells were permeabilized with 1% Triton X-100 for 25 min and then blocked with 2.5% (w/v) BSA in PBS for 1 h. PCNA was probed for using mouse anti-PCNA antibody (F-2, Santa Cruz) at a 1:250 dilution. 8-oxoGuanine was probed using for mouse anti-8-Oxoguanineantibody (MAB3560, Millipore Sigma) at a 1:250 dilution. DAPI (10 μM; Thermo Fisher Scientific) at 1:333 dilution was used for visualising DNA. Imaging was done using Olympus IX81 inverted fluorescence microscope with a Hamamatsu C9100-13 back-thinned EM-CCD camera and Yokogawa CSU ×1 spinning disk confocal scan head.

### siRNA knockdown

HL-60 cells (ATCC) were cultured in DMEM with 10% vol/vol FBS at 37 °C and 5% CO_2_. The cells were differentiated with 7% dimethylformamide (Sigma-Aldrich). The media supplemented with 7% dimethylformamide was replaced after 2 days. A mixture of siRNA and transfection reagent was prepared by mixing the two components by vertexing well and allowing the mixture to sit at room temperature for 10 min. On day 4, the siRNA (final concentration of 25 nM scrambled siRNA, APE1, or PARP1; Qiagen) and HiPerFect transfection reagent (final concentration of 2 μl per 100 μl, Qiagen) mixture were added to the cells. Following a 24 h knockdown, the cells were washed and resuspended in fresh RMPI without FBS. The PARP1 siRNA target sequence used was 5′-CCGAGAAATCTCTTACCTCAA-3′ with a sense strand of 5′-GAGAAAUCUCUUACCUCAATT-3′. The APE1 siRNA target sequence used was 5′-CAGGACAGAGCCAGAGGCCAA-3′ with a sense strand of 5′-GGACAGAGCCAGAGGCCAATT-3′.

To confirm knockdown, western blot was performed. For lysates, 2.5 × 10^5^ HL-60 cells were placed in Eppendorf tubes in a volume of 40 μl (following differentiation and 24 h knockdown) to ensure that there was equal loading of all samples. Samples were lysed with the addition of 10 μl of lysis buffer containing complete protease inhibitor mixture (Roche) supplemented with NaVO3 (5 mM), leupeptin (125 µM), pepstatin (125 μM), aprotinin (125 µM), NaF (125 mM), levamisole (5 mM), freshly prepared phenylmethylsulfonyl fluoride (5 mM), and 0.5% (w/v) Triton X-100. Samples were then sonicated thrice for 3 min using an aquasonic sonicator (VWR, model 50D) at maximal settings. Loading buffer (5×; 10 μl) containing Tris-HCl (125 mM, pH 6.8), 6% (w/v) SDS, 8% (v/v) β-mercaptoethanol, 18% (v/v) glycerol, 5 mM EDTA, 5 mM EGTA, leupeptin (10 μg/ml), pepstatin (10 μg/ml), aprotinin (10 μg/ml), NaF (10 mM), NaVO3 (5 mM), and levamisole (1 mM) was added to samples. Samples were then heated at 95 °C for 10 min on a heat block (Eppendorf). The samples were size-fractionated on a 12% (w/v) resolving and 5% (w/v) stacking bis-acrylamide gels for 25 min at 100 V and 30 min at 200 V. Using a wet transfer system, proteins were transferred from the gel onto a nitrocellulose membrane for 90 min The membrane was blocked with 5% (w/v) BSA in PBS containing 0.05% (w/v) Tween-20 buffer for 1 h at room temperature. The antibodies used were anti-APE1 (ab194; abcam) at 1:500, anti-PARP (#9542, Cell Signalling) at 1:500 and anti-GAPDH (ab9483; abcam). Secondary antibodies used were conjugated with horseradish peroxidase (HRP). HRP substrates used were enhanced chemiluminescent reagents. Membranes were imaged using a Li-Cor Odyssey FC Imaging System.

### In-cell ELISA

Cells (5 × 10^6^ cells per ml) were plated on a 96-well plate, and incubated with PMA or LPS for 2 h at 37 °C. The reactions were terminated with 4% (w/v) PFA (Sigma-Aldrich) overnight. Cells were permeabilized with 1% Triton X-100 for 25 min and then blocked with 2.5% (w/v) BSA in PBS for 1 h. 8-oxoG was probed for using mouse anti-8-Oxoguanineantibody (MAB3560, Millipore Sigma) at a 1:250 dilution. Plate was Imaging was done using Olympus IX81 inverted fluorescence microscope with a Hamamatsu C9100-13 back-thinned EM-CCD camera and Yokogawa CSU ×1 spinning disk confocal scan head. Fluorescence was measured using POLARstar OMEGA fluorescence plate reader (BMG Labtech; excitation = 485 nm, emission = 525 nm).

### Statistical analyses

Statistical analyses were performed using GraphPad Prism 7. One-Way ANOVA with Dunnett and Tukey’s posttests, Two-way ANOVA with Dunnett’s posttest and Student’s *t* test were performed as appropriate. Variance between groups compared is similar. Error bars in graphs represent ±SEM. A *p* value < 0.05 was considered to be statistically significant.
